# Rapid Detection of Foodborne Pathogenic Bacteria in Beef Using Surface-Enhanced Raman Spectroscopy

**DOI:** 10.3390/foods14193434

**Published:** 2025-10-07

**Authors:** Huixin Zuo, Yingying Sun, Mingming Huang, Yuqi Liu, Yimin Zhang, Yanwei Mao

**Affiliations:** 1 College of Food Science and Engineering, Shandong Agricultural University, Tai’an 271018, China; hxzuo@sdau.edu.cn (H.Z.); yysun1022@163.com (Y.S.); hades3709@126.com (M.H.); 18562322192@163.com (Y.L.); ymzhang@sdau.edu.cn (Y.Z.); 2 College of Intelligent Engineering, Taishan Science and Technology College, Tai’an 271018, China

**Keywords:** beef, foodborne pathogens, surface-enhanced Raman spectroscopy, chemometrics

## Abstract

Foodborne pathogenic bacteria in meat pose a serious threat to human health. Traditional detection methods for these bacteria are often time-consuming and labor-intensive. In this study, we applied surface-enhanced Raman scattering (SERS) combined with portable Raman spectroscopy as a rapid and convenient detection technique. SERS is a sensitive and fast method that enhances light scattering on rough metal surfaces. Silver nanoparticles (AgNPs) were used as SERS substrates to identify and analyze four pathogenic bacteria, including *Escherichia coli* (*E. coli*) O157:H7, *Salmonella typhimurium* (*S. typhimurium*), *Staphylococcus aureus* (*S. aureus*), and *Listeria monocytogenes* (*L. monocytogenes*), in beef. We optimized the detection conditions of AgNPs and established the limit of detection (LOD) for these four pathogenic bacteria in both pure culture and beef samples. The LODs were as low as 4–23 CFU/mL in beef samples, indicating high detection sensitivity. Linear discriminant analysis (LDA) was used to analyze the SERS spectra, yielding an accuracy of 91.7–97.3%. This study not only provides a rapid and portable detection method for pathogenic bacteria in beef but also overcomes the limitations of traditional methods that are often time-consuming and not suitable for on-site detection. However, the current study is limited to the detection of the four specific pathogenic bacteria, and further research is needed to expand the range of detectable pathogens and to improve the robustness of the detection models for more complex meat samples. Overall, this research demonstrates the potential of SERS combined with portable Raman spectroscopy as a powerful tool for the rapid detection of pathogenic bacteria in meat products, which could significantly enhance food safety monitoring and control.

## 1. Introduction

In recent years, there has been a significant global surge in food production and sales due to rapid economic growth and an accompanying rise in living standards. Unfortunately, this increase in food consumption has also resulted in a heightened prevalence of foodborne diseases, which have become a major public health concern worldwide [1]. The presence of foodborne bacteria such as *Escherichia coli* (*E. coli*) O157:H7, *Salmonella typhimurium* (*S. typhimurium*), *Staphylococcus aureus* (*S. aureus*), and *Listeria monocytogenes* (*L. monocytogenes*) in contaminated food or water poses a substantial risk to human health due to the severe illnesses they can cause [2,3]. Therefore, reliable and rapid bacterial detection has become an urgent need in the field of public health protection and food safety [4].

Nutrient-rich beef serves as an optimal medium for the proliferation of diverse microorganisms. Although cooking effectively eliminates most bacteria, the growing popularity of ready-to-eat foods has escalated the risk of pathogen exposure [5]. Consequently, extensive research has been conducted on the detection of pathogenic bacteria in beef products. For instance, Taylor et al. [6] employed techniques such as double-strength titanous hydroxide for pathogen concentration followed by meticulous DNA extraction and PCR assay processes to successfully identify *E*. *coli* O157:H7 in ground beef. Despite their high accuracy rates, these methods are time-consuming and do not facilitate rapid detection. Conversely, Meisel et al. [7] established a three-tier support vector machine (SVM) classification model based on Raman spectroscopy and investigated the isolation of pathogenic bacteria in beef samples. However, they solely utilized bacterial suspension with a concentration of 10^7^ CFU/mL as test samples without assessing detection sensitivity at lower concentrations. Given the limitations identified in this study and the need for improvement within this field, there is still a requirement to develop a rapid and sensitive method for detecting foodborne pathogens to ensure food safety [8].

Raman spectroscopy, due to its capability of providing insights into the concentration, structure, and interactions of biochemical molecules within cells and tissues, is a valuable tool for detecting and identifying foodborne pathogenic bacteria. As demonstrated by Mircescu et al. [9], Raman spectroscopy combined with spectral pre-processing and chemometrics can effectively differentiate between various bacterial genera and serotypes. Liu et al. [10] utilized surface-enhanced Raman scattering (SERS) combined with chemometrics to rapidly identify different phages in spice powders. However, Raman spectroscopy encounters limitations such as low sensitivity and resolution that may impede its effectiveness in bacterial detection and identification [11]. To overcome these limitations, SERS has been developed. SERS employs rough metal surfaces like silver, gold, and copper to significantly enhance Raman signals—up to 7 orders of magnitude—compared to standard Raman scattering [12]. This advancement has proven particularly beneficial in microbiology for bacterial classification and identification, as noted by Pahlow et al. [13]. Furthermore, Wang et al. [14] established a classification model using SERS that allows for differentiation between *E*. *coli* and *Staphylococcus* spp., based on their Gram types and separation of Gram-positive and Gram-negative bacteria. Akcinar et al. [15] developed a method utilizing magnetic gold nanoparticles for pre-enrichment of *L*. *monocytogenes*, resulting in a capture efficiency of 75% and an impressive detection limit as low as 12 CFU/mL.

Despite the significant potential of SERS technology in detecting foodborne pathogens, current research has primarily focused on pure cultures under ideal conditions. There is a noticeable lack of studies investigating the direct detection of these pathogens within complex matrices such as beef products. To address these challenges, our study aims to investigate whether SERS, in conjunction with chemometric methods, can be effectively utilized for rapid and sensitive detection of common foodborne pathogens in beef samples. A key objective of our research is to determine the limit of detection (LOD) for SERS and enhance bacterial identification precision through linear discriminant analysis (LDA). We propose that leveraging the Raman signal enhancement provided by SERS, establishing LOD values, and employing LDA will facilitate accurate identification and differentiation of these pathogens both in pure cultures and within the more intricate environment found in beef samples.

## 2. Materials and Methods

### 2.1. Test Strains and Chemicals

Test strains of *E. coli* O157:H7, S2, *S. typhimurium* ATCC 14028, *S. aureus* ATCC 25923, and *L. monocytogenes* ATCC 19115 provided by the Laboratory of Beef Processing and Quality Control, College of Food Science and Engineering, Shandong Agriculture University. Among them, *E. coli* and *S. typhimurium* are Gram-negative bacteria, while *S. aureus* and *L. monocytogenes* are Gram-positive bacteria.

Silver nitrate (99%), sodium citrate (99%) and ethanol were purchased from Sinopharm Chemical Reagent Co. Ltd. R6G was purchased from Beijing Solebo Technology Co., Ltd. (Beijing, China) All other chemicals were of analytical grade and used without further purification.

### 2.2. Culture of Single and Mixed Bacteria

*E. coli* O157:H7, *S. typhimurium*, *S. aureus*, and *L. monocytogenes* cryopreserved at −80 °C were inoculated into fresh brain heart infusion (BHI) medium and incubated with shaking (200 r/min, 37 °C) for 18 h. The activated bacteria suspension was centrifuged at 6000× *g* rpm at 4 °C for 10 min to remove the medium and washed 3 times with sterilized water. Finally, the bacteria suspension was re-suspended in sterile saline (bacterial count at about 8 log CFU/mL), diluted in 10 gradients at a ratio of 10 times, and used as the test samples for LOD analysis. For the research on mixed bacteria, different pathogenic bacterial solutions were mixed in equal volumes, and the resulting mixed bacterial solution was used as the sample for the mixed bacteria experiment.

### 2.3. Preparation of AgNPs

AgNPs were prepared following the Lee-Meisel method of sodium citrate reduction [16]. To this end, silver nitrate (0.018 g) was dissolved in ultrapure water (100 mL) to form a solution. This solution was brought to a boil on an electric stove. Then, 2 mL of a 3% sodium citrate solution was added, and the mixture was heated under reflux on a constant temperature magnetic stirrer. Maintain boiling and stirring for 60 min to ensure complete decomposition of silver nitrate and sodium citrate in the solution, thereby minimizing background interference in Raman spectroscopy. This process results in the formation of a silver nanosol containing AgNPs. The prepared silver nanosol was naturally cooled to room temperature and after centrifugal washing with ultrapure water 3 times, the silver nanosol was re-suspended with ultrapure water of equal volume and stored in the dark at 4 °C. The complete mixing of the silver nanosol before use is crucial due to the precipitation of AgNPs during storage.

### 2.4. Physico-Chemical Characterization of the SERS-Enhanced Substrate

#### 2.4.1. Ultraviolet–Visible (UV-Vis) Spectroscopy Characterization of AgNPs

To assess the optical properties and stability of the silver nanosol, which are crucial for SERS substrate performance. UV-Vis (Nano Drop™ 1000, Thermo Fischer, Waltham, MA, USA) spectrophotometry was performed on the prepared silver nanosol with the scanning range set at 300–700 nm and the scanning speed set to medium (600 nm/min). For each strain, different concentrations (ranging from 10 CFU/mL to 108 CFU/mL) were used for regression contamination. Three parallel samples were prepared for each concentration, and each parallel sample was scanned 10 times to obtain Raman spectra. A total of 3 repeated experiments were conducted, and the recovery rate and detection limit (LOD) of SERS for detecting pathogenic bacteria in meat products were calculated.

#### 2.4.2. Scanning Electron Microscopy (SEM) Characterization of AgNPs

The silver nanosol was centrifuged at 7000 rpm for 5 min at room temperature, the supernatant was discarded, and the precipitate was dissolved and diluted with ultrapure water until it was clear and transparent. The sample was dripped on a 5 × 5 mm silicon wafer and dried naturally to form a film. The dried sample was then coated with gold using a vacuum sputter coater. The sample was placed on a sample stage about 10–15 cm away from the evaporation source, rotated for uniformity and evenly sputtered with gold (10 kV, 60 s). When the coating was done, the sample was observed under the scanning electron microscopy (SEM). After the gold coating process was completed, the sample was then carefully examined using SEM to characterize the morphology and distribution of the AgNPs.

#### 2.4.3. Setting of the Acquisition Parameters for Raman Spectroscopy

(1) Settings for the acquisition parameters of the portable Raman spectrometer: The instrument used for scanning the Raman spectrum is a portable Raman spectrometer (Raman Pro, B&WTek, Newark, DE, USA), equipped with a 785 nm laser diode. The laser power is at the maximum of the instrument (i.e., 100%), the integration time is 20 s, the average scanning times are 2, and the range of Raman shift is 400–1800 cm^−1^.
(2) Settings for the acquisition parameters of the laser confocal micro-Raman spectrometer: The excitation wavelength is 532 nm, the laser power is 14 mW, a 20× objective lens is used, the integration time is 1 s, the integration times are 10, and the range of Raman shift is 400–1800 cm^−1^.

#### 2.4.4. SERS-Enhanced Effect Test of AgNPs

In order to evaluate the SERS-enhanced effect of the substrate, R6G dye molecule was used as a probe to test the enhancement performance of the prepared AgNPs. R6G solution with a concentration of 1000 μg/mL was prepared using 0.85% saline solution, mixed with silver nanosol at a fixed ratio, and the Raman spectroscopy was performed on R6G-AgNPs and R6G separately. For each sample, three parallel samples were prepared and the measurement was repeated 3 times. Finally, the enhancement factor (EF) was calculated in combination with the obtained Raman spectra to determine the enhancement effect of AgNPs.

In practical SERS detection, the following equation was used to estimate the theoretical EF with the measured values in a quick and easy manner.

(1) $$EF=\frac{\left[I_{SERS}/N_{SERS}\right]}{\left[I_{NR}/N_{NR}\right]}=(\frac{I_{SERS}}{I_{NR}})(\frac{C_{NR}S_{SERS}}{C_{SERS}S_{NR}})$$

where

*I_SERS_*—the intensity of the SERS signal;

*N_SERS_*—the number of molecules adsorbed by the enhanced substrate;

*I_NR_*—the intensity of standard Raman signal;

*N_NR_*—the number of molecules in standard Raman detection region;

*C_NR_*—the concentration of the sample in standard Raman detection;

*C_SERS_*—the concentration of the analyte in SERS detection;

*S_NR_*—the unfolded area of the sample in standard Raman detection;

*S_SERS_*—the unfolded area of the sample in SERS detection.

#### 2.4.5. Stability Evaluation of the Stored Silver Nanosol over Time

In order to evaluate the stability of the silver nanosol over time, the prepared silver nanosol was stored at 4 °C away from light and required to be fully mixed before each use. Using *S. typhimurium* as the detection target, for each day from day 1 to day 15, an equal volume of silver nanosol was taken and mixed with the bacteria suspension obtained in Section 2.2 (5 parallel samples per day), and their Raman spectra were acquired following the same procedure.

### 2.5. Determination of SERS Limit of Detection (LOD) for Different Foodborne Pathogens

To calculate the limit of detection (LOD) of SERS for different foodborne pathogens, we used the formula LOD = *KS_0_*/*S*, where *K* is a numerical factor chosen according to the confidence level desired (*K* = 3), *S_0_* is the standard deviation (S.D.) of *n* blank measurements (*n* = 10) and *S* is the slope of the standard curve [17].

### 2.6. Preparation of Beef Samples

A commercially available fresh vacuum-packaged beef was used to prepare the meat identification samples. From these materials, 2.0 g of fresh beef was taken. Both the front and back sides were exposed to ultraviolet light for 30 min each to eliminate interference from contaminants. 18 mL of sterile water was then added to it. Subsequently, the beef was ground and mixed to form a meat paste. The volume is approximately 20 mL. An equal volume of bacteria suspension was added, fully mixed with the suspension, and shaken at room temperature for 10 min for sufficient binding of the homogenized ground beef and bacteria. After this, 5 mL of the ground beef-bacteria suspension was filtered using a 5 μm sterile filter to remove the beef residue and collect the juice [7]. The obtained juice was then centrifuged at 6000× *g* rpm for 3 min at 4 °C, before the supernatant was discarded and the precipitate was washed 3 times with sterile saline. Finally, the precipitate was re-suspended in 2.5 mL of sterile saline and used as the beef sample for SERS detection. The entire scanning process was completed within 1 min.

### 2.7. Determination of LODs of SERS for Pathogenic Bacteria in Beef Samples

To obtain the LODs of Raman spectroscopy for the four pathogenic bacteria in beef samples, the following steps were performed. Initially, 2.0 g of fresh ground beef was irradiated on both sides with UV light for 30 min each to eliminate any existing microbial contamination. The irradiated ground beef was then homogenized in 18 mL of sterile water to create a beef slurry. Different concentrations (10~108 CFU/mL) of each of the four pathogenic bacteria (*Salmonella typhimurium*, *Escherichia coli*, *Staphylococcus aureus*, and *Listeria monocytogenes*) were prepared by serial dilution. Each beef slurry was spiked with one of the prepared bacterial suspensions to simulate the bacterial environment in meat. After spiking, the beef slurry was mixed thoroughly and incubated at room temperature for 10 min to allow the bacteria to adhere to the beef particles. The beef slurry was then filtered through a 5 μm sterile filter to remove larger meat particles, and the filtrate was collected. The filtrate was centrifuged at 6708× *g* for 3 min at 4 °C to pellet the bacteria. The pellet was washed three times with sterile physiological saline to remove any residual beef particles and debris. The washed bacterial pellet was resuspended in 2.5 mL of sterile physiological saline to create the final beef sample for SERS analysis. This final beef sample was mixed with an equal volume of silver nanosol to form bacteria-AgNPs complexes. The complexes were incubated at room temperature for 30 min to ensure proper binding of AgNPs to the bacterial surface. SERS spectra were collected using the portable Raman spectrometer (Raman Pro, B&WTek, LLC, USA) with the parameters set as described in Section 2.4. The collected SERS spectra were preprocessed using the SNV method to reduce baseline drift and enhance spectral features. Linear regression equations and R^2^ values were calculated between the Raman signal intensity for each foodborne pathogen at different concentrations and the Log10 value of their concentrations. The LODs for the four pathogenic bacteria in beef samples were determined using the same method described in Section 2.5, where LOD = 3 N/K, with N being the standard deviation of the blank sample Raman intensity and K being the slope of the linear regression equation.

### 2.8. SERS Measurements

SERS signals were acquired using the portable Raman instrument (Raman Pro, B&WTek, USA) with a 785 nm laser diode, a laser power of 300 mW and an integration time of 20 s. The acquisition range of Raman displacement is 400–1800 cm^−1^. Each sample was repeated no less than 10 times.

### 2.9. Statistical Analysis

The average spectrum for each sample was calculated from the spectral data acquired from each sample after pre-processing. The spectra were trimmed to the range of 400–1800 nm. Each spectrum was smoothed by a Savitzky-Golay filter with a polynomial order of 2 and a window size of 11. Baseline correction was performed using the asymmetric least squares method. The spectra were then normalized by standard normal variate (SNV) transformation to reduce the scattering effects. The supervised pattern recognition method, LDA, was employed to analyze the average spectra. The optimal number of latent variables (LVs) for LDA was determined by cross-validation using the leave-one-out method. The quality of the LDA models was verified by calculating the classification accuracy, sensitivity and specificity on both the calibration and validation sets [18]. For all the above procedures, the spectral data were imported into Matlab R2016a (Mathworks, Inc., Natick, MA, USA) for analysis and processing, and the software OriginPro 2019b (OriginLab Corporation, Northampton, MA, USA) was used for graphing.

## 3. Results and Discussion

### 3.1. Physico-Chemical Characterization of AgNPs as the SERS-Enhanced Substrate

#### 3.1.1. UV-Vis Spectrophotometry of AgNPs

**Figure 1:**
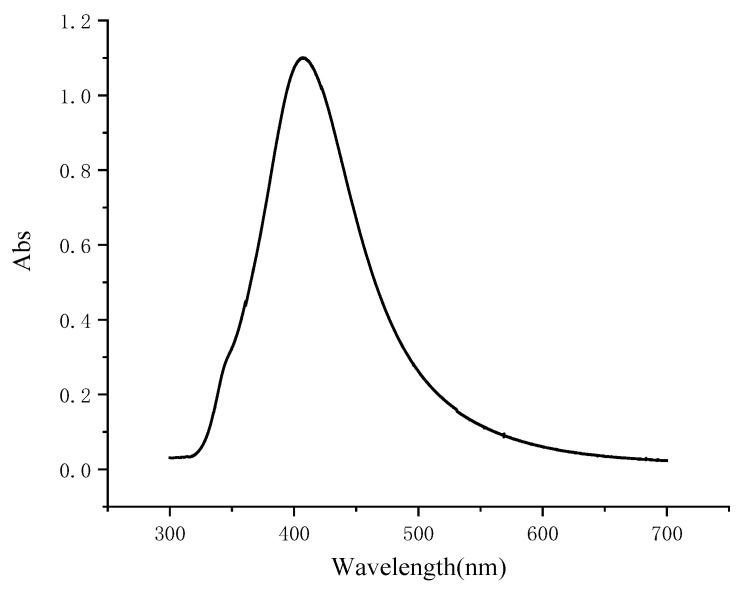
UV-Vis spectrum of silver nanosol.

In this experiment, the silver nanosol obtained through the reduction of silver nitrate with sodium citrate had high purity as demonstrated by the absorption peaking around 410 nm (Figure 1). This absorption peak corresponds to the localized surface plasmon resonance (LSPR) phenomenon of AgNPs, which is influenced by their size, shape, and distribution [19]. Previous studies have reported similar absorption peaks for AgNPs synthesized by different methods [20]. Therefore, the absorption peak at 410 nm can be used as a reliable indicator of the quality and performance of the silver nanosol for SERS detection. Moreover, the stirring and heating steps under reflux were effective in improving the stability and prolonging the storage life of the prepared nanosol, as evidenced by the smooth and even spectrum line. Therefore, the prepared silver nanosol met the requirement for SERS detection of foodborne pathogenic bacteria.

#### 3.1.2. SEM Detection of AgNPs

**Figure 2:**
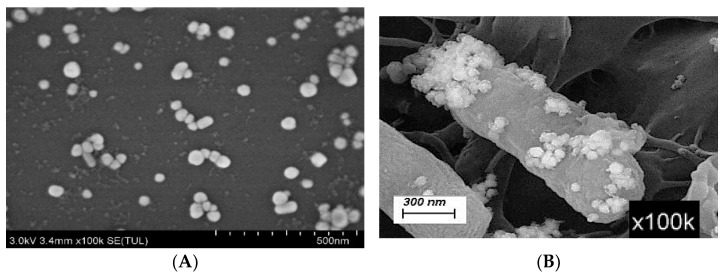

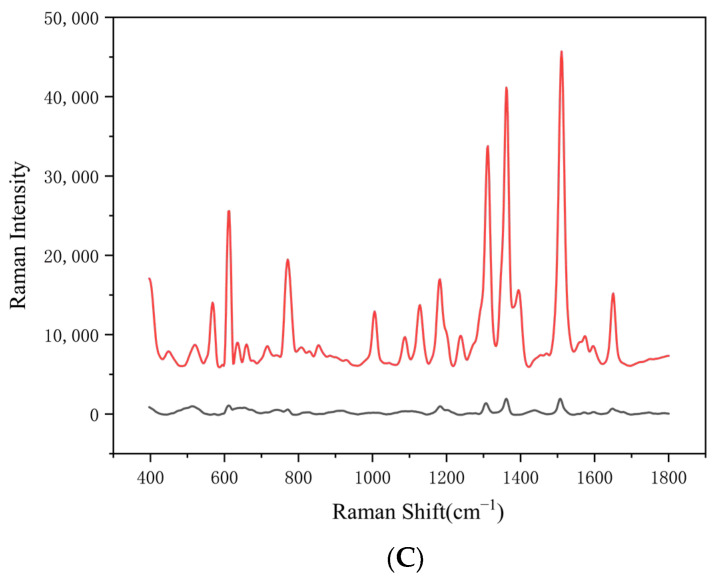
SERS enhanced performance test of AgNPs. The SEM image of silver nanosol was shown in (**A**). Bacterial cells mixed with AgNPs were shown in (**B**). Effect of silver nanosol on R6G Raman spectral intensity was shown in (**C**). In (**C**), the red line represented the SERS spectrum of R6G-AgNPs (0.1 μg/mL), while the black line represented the standard Raman spectrum of R6G (1000 μg/mL).

To assess the SERS-enhanced performance of AgNPs, SEM, R6G dye molecule and Raman spectroscopy were used. The prepared AgNPs had a near-round shape, a uniform size, and a strong attachment to the bacterial cells, which resulted in a high enhancement factor of R6G of about 2.06 × 10^5^. This demonstrates that the prepared silver nanosol can effectively enhance the Raman signal of the target analytes and enable the SERS detection of pathogenic bacteria. As shown by Figure 2A the SEM test results of AgNPs, the AgNPs are well dispersed without any large agglomerates. This is consistent with the results of Chen et al. [21], which indicate that the prepared AgNPs can serve as ideal enhancement substrates for the SERS detection of pathogenic bacteria as the AgNPs were in close contact with bacterial cells and were mostly attached to the surface of bacterial cells (Figure 2B). The average particle size was 170 ± 7 nm, as calculated by Nano Measure software v1.2.5. AgNPs were aggregated and formed Ag nanoparticles clusters on the surface of bacterial cells. Therefore, bacterial cells were coated by these clusters that produced more hot spots on the interfaces of the bacterial cell wall, resulting in the enhancement of the Raman signal.

#### 3.1.3. Evaluation of SERS-Enhanced Performance of AgNPs

The relatively high Raman signal intensities at 1312 cm^−1^, 1379 cm^−1^, and 1509 cm^−1^ as can be seen in Figure 2C, were identified as the characteristic peaks of R6G, while the highest Raman intensity was found at 1509 cm^−1^. Therefore, the Raman peak at this location was chosen for the EF calculation. The relative intensity of the characteristic peak of the standard Raman spectrum of R6G with a concentration of 1000 μg/mL at 1509 cm^−1^ was 1918.99, while the intensity of the characteristic peak of the SERS spectrum of R6G with a concentration of 0.1 μg/mL enhanced by AgNPs was 39517.7. The unfolded area of the analyte was the same in both SERS and standard Raman detection. These measurements yielded an enhancement factor of R6G of about 2.06 × 10^5^, indicating a significant enhancement effect of the prepared silver nanosol on the SERS signal. This result indicates that the prepared silver nanosol had a significant enhancement effect on the SERS signal. This agrees with previous studies such as Zeiri et al. [22] who also found that an enhanced Raman signal could be obtained by SERS measurement of bacteria samples treated with AgNPs.

#### 3.1.4. Stability Evaluation of the Stored Silver Nanosol over Time

**Figure 3:**
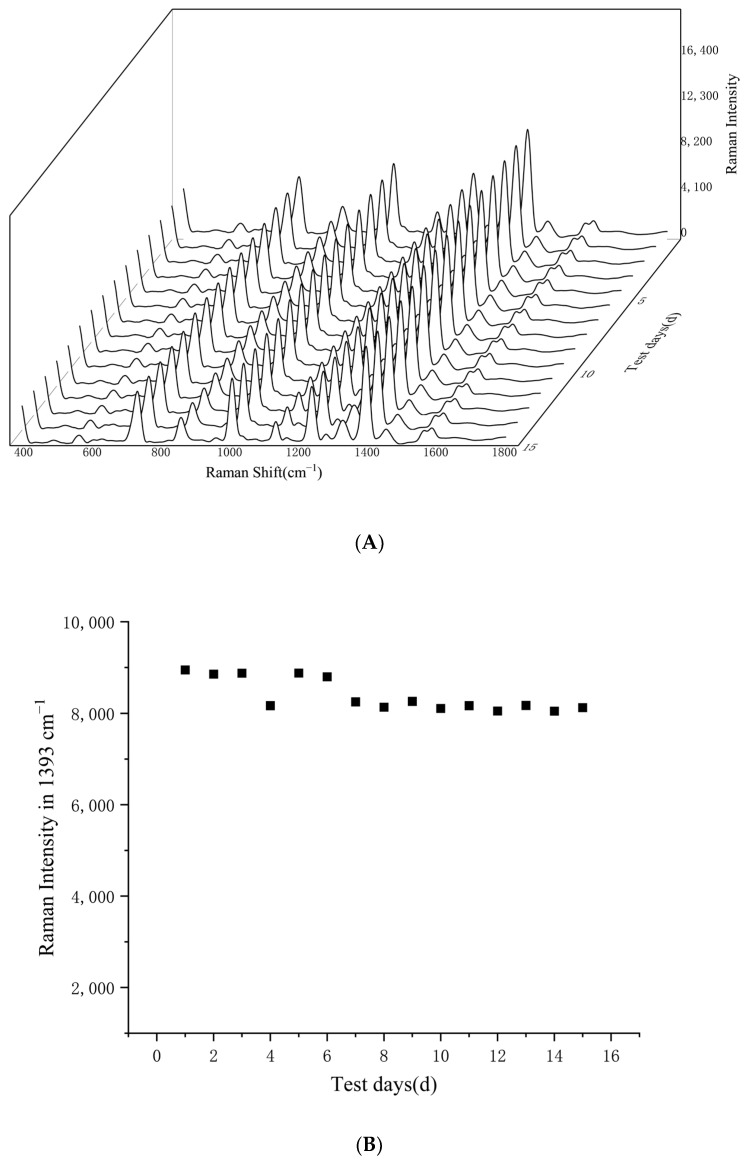
Stability evaluation of the silver nanosol throughout 15 days. The SERS spectra of bacteria-AgNPs collected were shown in (**A**). Variation in Raman strength at 1393 cm^−1^ was shown in (**B**).

Overall, the prepared silver nanosol had good stability and reproducibility throughout 15 days, with a relative standard deviation (RSD) of Raman intensity at 1393 cm^−1^ of 4.26%. In this research, *S. typhimurium* was used as the detection target for stability evaluation of silver nanosol over time, and the SERS spectra of *S. typhimurium*-AgNPs conjugates were collected for 15 consecutive days (5 parallel samples per day). As can be observed from the average spectra shown in Figure 3A, the Raman signals of *S. typhimurium* were relatively strong at the shifts of 726 cm^−1^, 1001 cm^−1^, 1235 cm^−1^, and the most prominent peak at 1393 cm^−1^. This peak was used as a reference for comparison of the spectra signal collected for 15 consecutive days and as shown in Figure 3B, the Raman signals remained stable throughout the 15 testing days, with the obtained Raman spectra showing high homogeneity. Since this research can achieve rapid detection of foodborne pathogenic bacteria within 2 h, the proven stability of the prepared colloidal silver within 15 days indicates its good applicability to this research. Compared to other conventional methods for detection of pathogens, such as culture analysis and metabolic tests, which may take several days to a week [23], the SERS-based method can provide faster and more accurate results.

### 3.2. LOD Analysis of SERS Detection for 4 Foodborne Pathogenic Bacteria in Pure Culture

**Figure 4:**
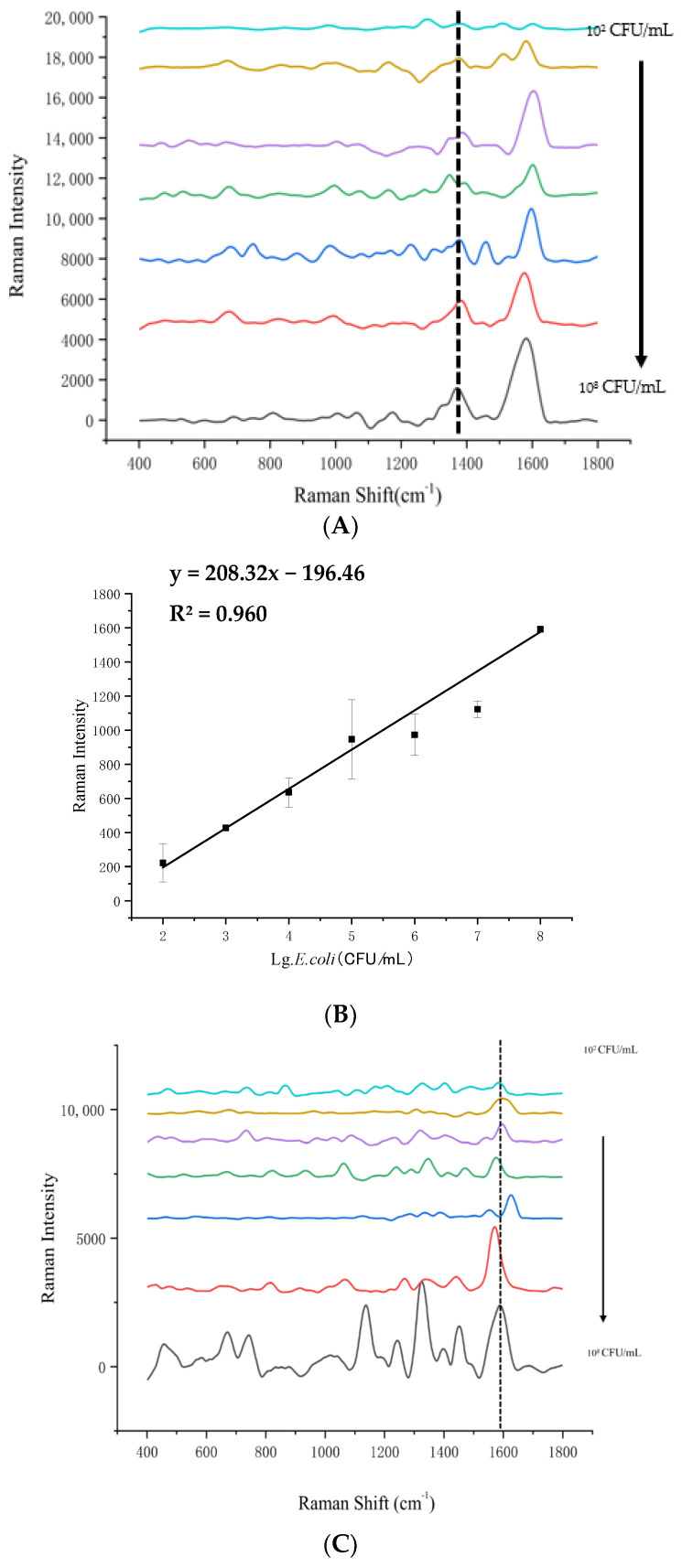

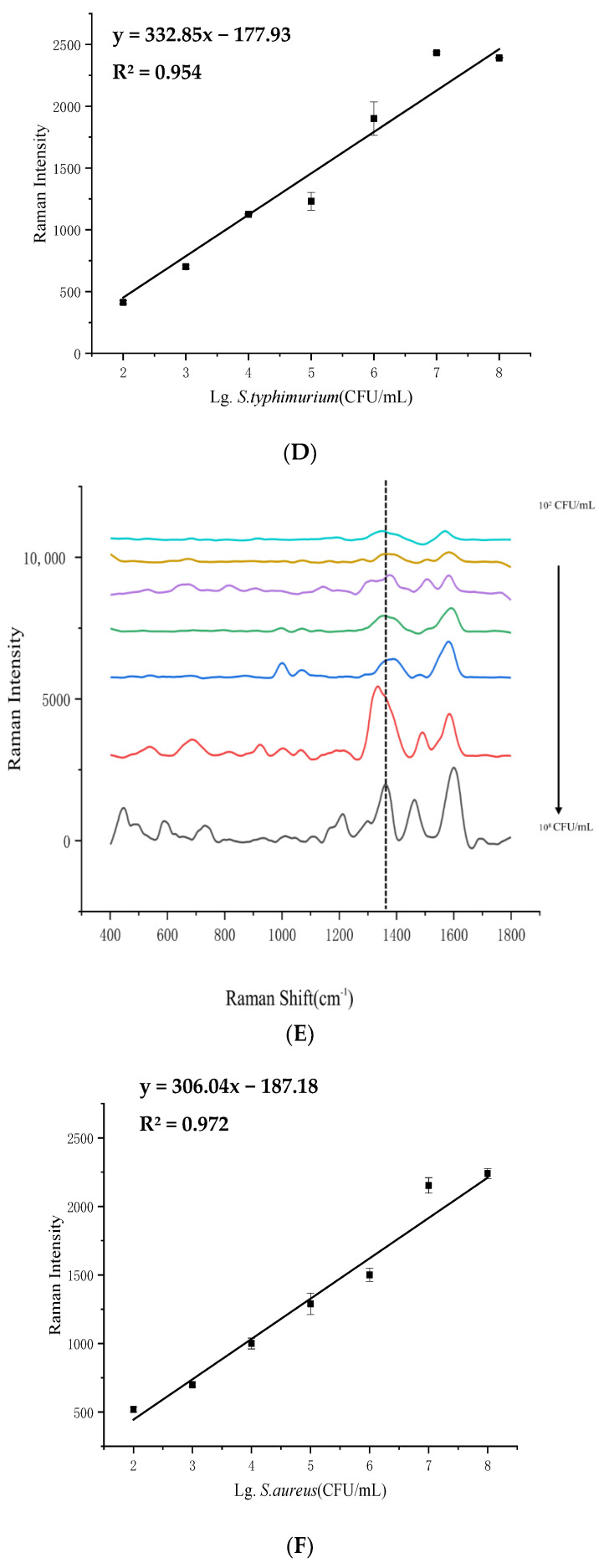

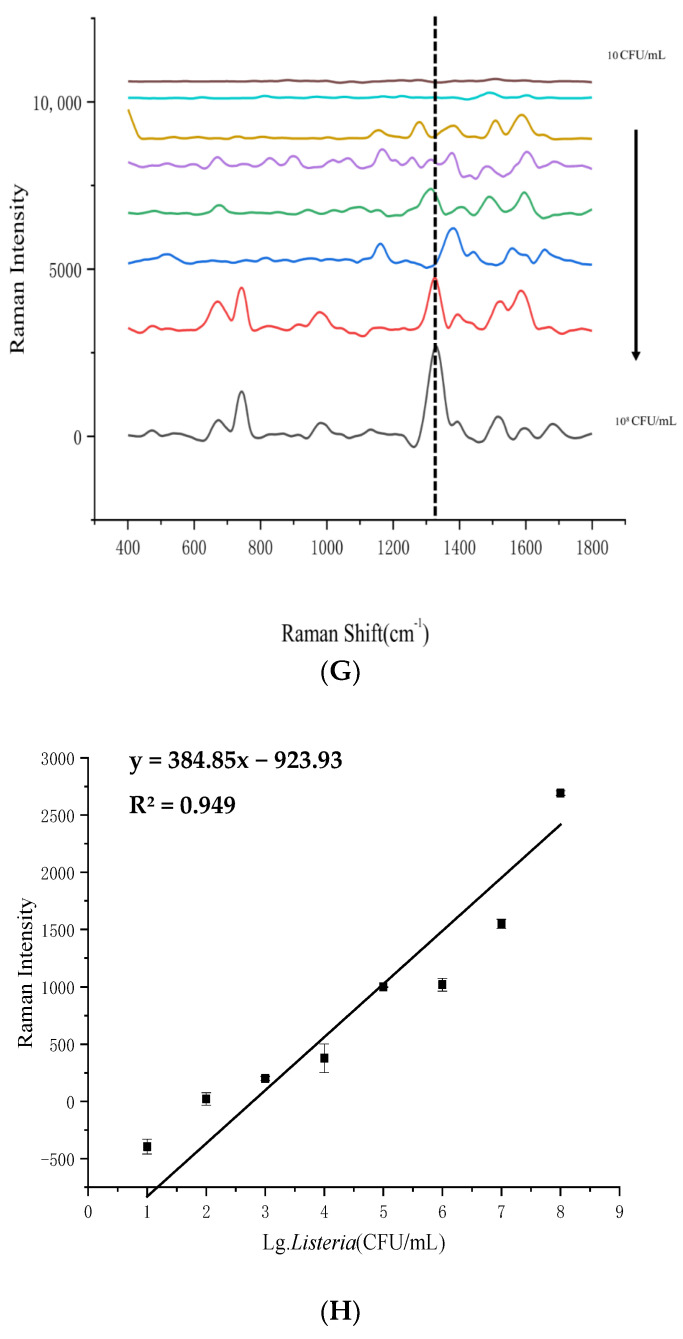
Raman spectra of different concentrations of bacteria in pure culture (**A**,**C**,**E**,**G**) and linear relationship between the Raman signal intensity and bacteria concentrations (**B**,**D**,**F**,**H**). The different colors in each plot represent different concentrations of bacteria (**A**,**C**,**E**,**G**). *E. coli* O157:H7 is shown in (**A**,**B**). *S. typhimurium* is shown in (**C**,**D**). *S. aureus* is shown in (**E**,**F**). *L. monocytogenes* is shown in (**G**,**H**).

The slope of the standard curve was obtained by performing a linear regression analysis on the Raman signal intensity of each foodborne pathogen at different concentrations and the Log_10_ value of their concentrations and the R^2^ value was also calculated to assess the goodness of fit of the linear model, as shown in Figure 4.

The Raman intensity of *E. coli* O157:H7 and *S. typhimurium* was measured across concentrations ranging from 10^2^ to 10^8^ CFU/mL. Both are Gram-negative rods that can cause foodborne infections, particularly in beef, beef burgers, and unpasteurized milk [24,25,26,27]. However, they exhibit different characteristic peaks in the Raman spectrum, which can be used for differentiation. Figure 4A shows that the signal intensity at the Raman shift 1350 cm^−1^ increased with the concentration of *E. coli* O157:H7. This peak may be attributed to the C-N stretching vibration of proteins or nucleic acids in *E. coli* cells [28]. Figure 4B shows a good linear relationship between the Raman signal intensity at 1350 cm^−1^ and the Log_10_ value of the concentrations of *E. coli* O157:H7, with a linear regression equation of y = 208.32x − 15.8, R^2^ = 0.9597 (x = Log concentration of *E. coli*, y = Raman signal intensity at 1350 cm^−1^). The limit of detection for *E. coli* O157:H7 was calculated as LOD = 16 CFU/mL. This result is comparable to that of Guven et al. [29], who combined immunomagnetic separation and SERS to determine *E. coli* O157:H7, showing a good linear relationship between bacterial concentration and SERS signal intensity within the range of 10~10^4^ CFU/mL (R^2^ = 0.992) and a detection limit of 8 CFU/mL. However, our method does not require any pre-enrichment or separation steps, which can reduce the time and cost of detection.

The signal intensity at the Raman shift 1520 cm^−1^ increased with the concentration of *S. typhimurium I* (Figure 4C), which may be related to the biofilm formation of *Salmonella*, or at least one of its components, as some studies have found that it is a characteristic feature of *Salmonella* biofilm [30]. Furthermore, a good linear relationship between the Raman signal intensity at 1520 cm^−1^ and the Log_10_ value of the concentrations of *S. typhimurium* (Figure 4D), with a linear regression equation of y = 363.43x − 361.43, R^2^ = 0.9616 (x = Log concentration of *S. typhimurium*, y = Raman signal intensity at 1520 cm^−1^). The limit of detection for *S. typhimurium* was calculated as LOD = 9 CFU/mL and was similar to that of Kögler et al. [28], who successfully identified *E. coli* and *Salmonella* by using gold nanoparticles as a substrate for SERS spectrum scanning, with a detection limit of 10 CFU/mL for both bacteria. However, the method in the current study was also able to differentiate between *E. coli* and *Salmonella* based on their distinct peaks at 1350 cm^−1^ and 1520 cm^−1^, respectively, which could improve the accuracy and specificity of detection. No further peaks that have been reported to be associated with *Salmonella* biofilm, such as 1330 cm^−1^, 1030 cm^−1^ and 875 cm^−1^ were observed, which may indicate that our method is not sensitive enough to detect these features. Therefore, further research is warranted to clarify the origin and significance of these peaks in relation to *Salmonella* biofilm.

The method was also tested on two Gram-positive bacteria, namely *S. aureus* and *L. monocytogenes* [31,32]. As shown in Figure 4E–H) the SERS of these bacteria at concentrations ranging from 10^1^ to 10^8^ CFU/mL have characteristic peaks in the Raman spectrum around 1330 cm^−1^ and 1325 cm^−1^, respectively, which could be used to identify them.

For *S. aureus*, the signal intensity at 1330 cm^−1^ increased with the concentration of the bacteria solution (Figure 4E), which may be related to the phenylalanine ring breathing mode in *S. aureus* cells [33]. Although Figure 4F shows a good linear relationship between the Raman signal intensity at 1330 cm^−1^ and the Log_10_ value of the concentrations of *S. aureus* (y = 306.04x − 187.18, R^2^ = 0.972, where x = Log concentration of *S. aureus*, y = Raman signal intensity at 1330 cm^−1^), the limit of detection for *S. aureus* was calculated as LOD = 11 CFU/mL, which is lower than that of Zhang et al. [33]. This may be due to differences in methods as Zhang et al. [33] used gold nanoparticles modified with Raman molecules and aptamer to capture the tested bacteria and achieved a detection limit of 35 CFU/mL for *S. aureus*. Unlike their method, the method used in the current study could simplify the testing process and reduce costs, making detection of Staphylococcus aureus in meat samples more efficient and economical.

For *L. monocytogenes*, the signal intensity at 1325 cm^−1^ increased with the concentration of the bacteria suspension, as shown in Figure 4G. This peak may be attributed to the C-H deformation vibration of lipids or proteins in *L. monocytogenes* cells [15]. Figure 4H shows a good linear relationship between the Raman signal intensity at 1325 cm^−1^ and the Log_10_ value of the concentrations of *L. monocytogenes*, with a linear regression equation of y = 384.85x − 923.93, R^2^ = 0.9148 (x = Log concentration of *L. monocytogenes*, y = Raman signal intensity at 1325 cm^−1^). The limit of detection for L. monocytogenes was calculated as LOD = 9 CFU/mL, which is similar to that of Akcinar et al. [15], who enriched *L. monocytogenes* in samples by magnetic gold nanoparticles modified with antibodies and obtained a detection limit of 12 CFU/mL for *L. monocytogenes*. Unlike Akcinar et al. [15], the current method does not involve antibodies, which can increase the risk of false positives and decrease the stability of detection due to possible degradation or denaturation of antibodies. Therefore, this method can effectively detect *S. aureus* and *L. monocytogenes* in meat samples and prevent foodborne outbreaks caused by these bacteria.

### 3.3. LOD Analysis of 4 Pathogenic Bacteria in Beef Samples

Results from the recovery rates calculated by using the equation: “recovery% = detection concentration/addition concentration × 100%” [33], are shown in Table 1.

The recovery rates of *E. coli* O157:H7, which was added at concentrations of 10, 10^2^, and 10^3^ CFU/mL to the beef samples, were 90.51%, 110.17%, and 100.95%, respectively, with the RSD lower than 1.21%. Similarly, the recovery rates of *S. typhimurium*, which was added at concentrations of 10, 10^2^, and 10^3^ CFU/mL to the beef samples, were 92.98%, 96.00%, and 100.97%, respectively, with the RSD lower than 2.37%. For *S. aureus*, which was added at concentrations of 10, 10^2^, and 10^3^ CFU/mL to the beef samples, the recovery rates were 91.44%, 108.87%, and 101.37%, respectively, with the RSD lower than 2.88%. Finally, the recovery rates of *L. monocytogenes*, which was added at concentrations of 10, 10^2^, and 10^3^ CFU/mL to the beef samples, were 107.61%, 96.64%, and 96.10%, respectively, with the RSD lower than 3.08%. Taken together, the data showed that the recovery rates of the 4 meat-borne pathogenic bacteria in beef samples ranged from 90.51% to 110.17%, with an average recovery rate of 99.47%. This suggests that the method adopted in this research can detect pathogenic bacteria in beef with accurate and reliable results. This has further validated the fitness of SERS for the detection of the 4 pathogenic bacteria in meat products. However, there are still some challenges for industrial applications of this method, such as the need for portable and powerful SERS devices, which should be addressed by future research.

**Figure 5:**
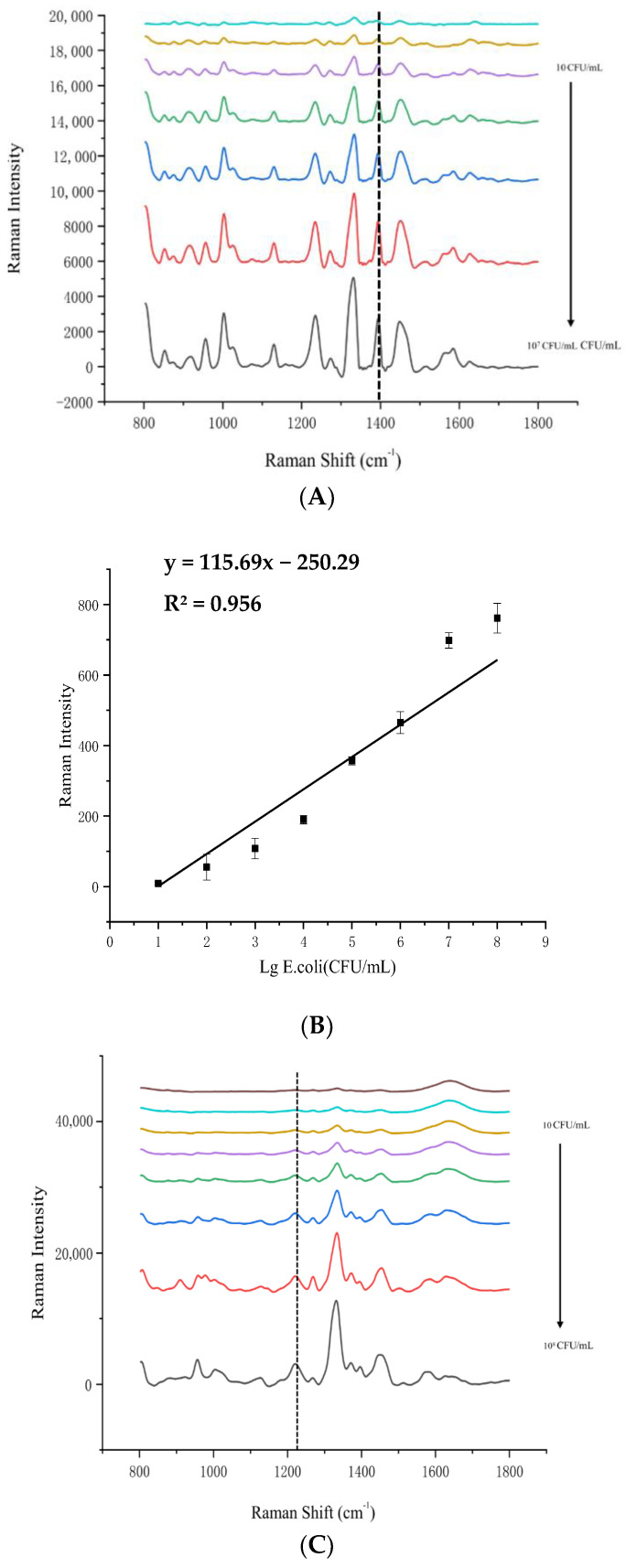

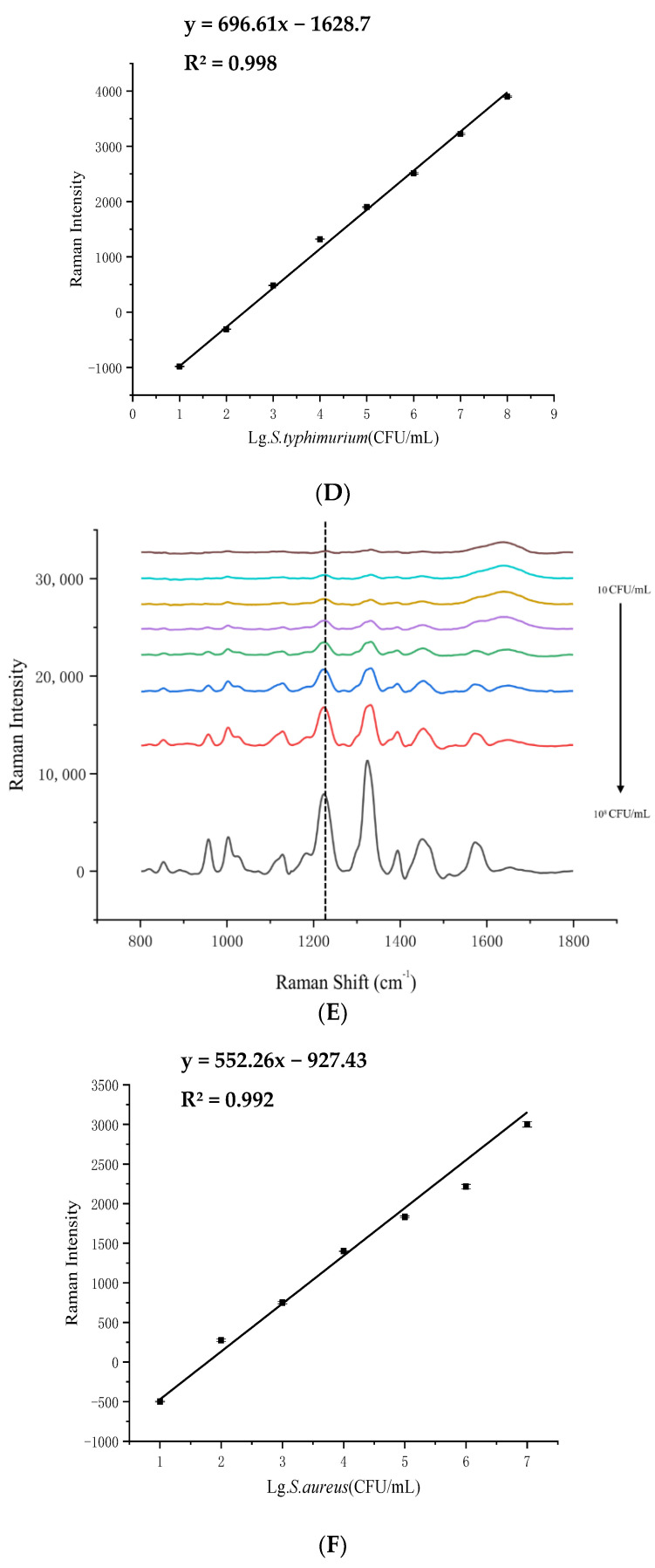

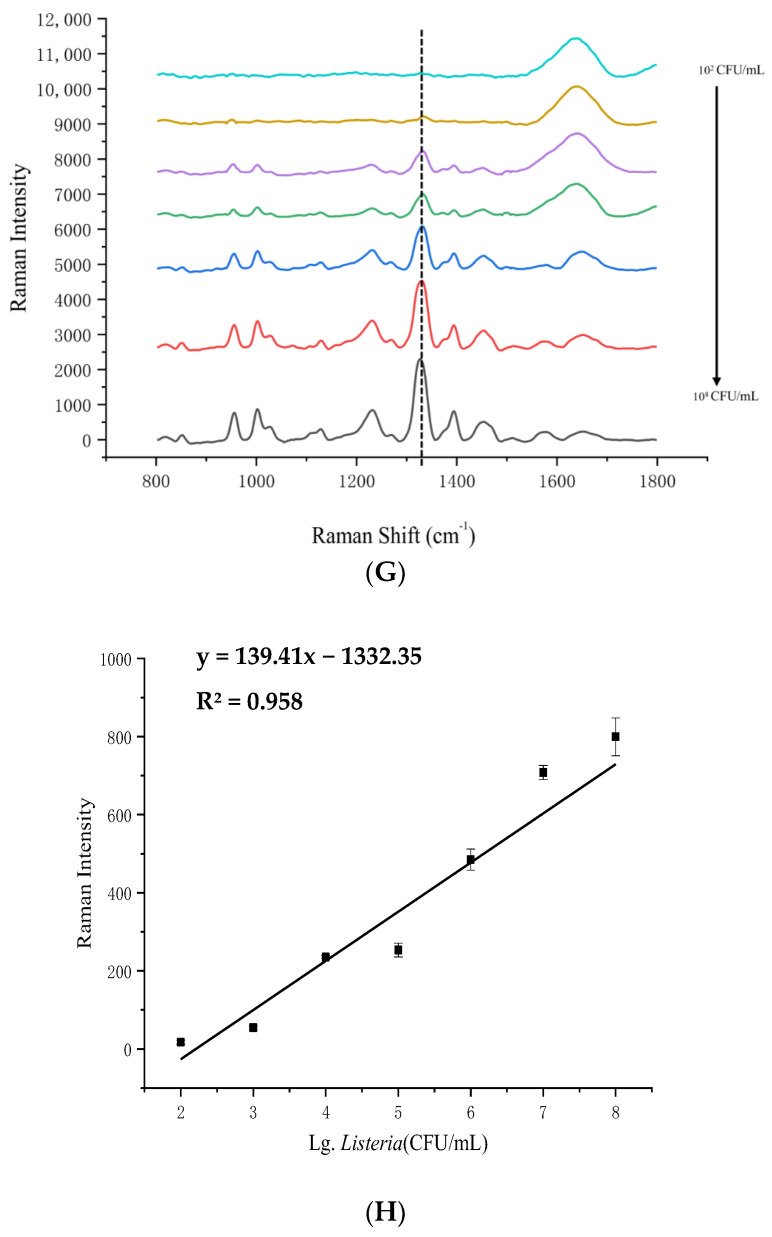
Raman spectra of different concentrations of bacteria in beef samples (**A**,**C**,**E**,**G**) and the linear relationship between Raman signal intensity and bacteria concentrations (**B**,**D**,**F**,**H**). The different colors in each plot represent different concentrations of bacteria (**A**,**C**,**E**,**G**). *E. coli* O157:H7 are shown in (**A**,**B**). *S. typhimurium* are shown in (**C**,**D**). *S. aureus* are shown in (**E**,**F**). *L. monocytogenes* are shown in (**G**,**H**).

Measuring the SERS of *E. coli* O157:H7, *S. typhimurium*, *S. aureus*, and *L. monocytogenes* at different concentrations ranging from 10^1^ to 10^8^ CFU/mL. Figure 4 illustrates the outcomes from pure bacterial cultures, whereas Figure 5 depicts the results from bacteria within beef samples. This demonstrates that our method can detect bacteria in complex matrices without interference from other components. Figure 5A,C,E,G show the signal intensities at the Raman shifts of 1320 cm^−1^, 1337 cm^−1^, 1250 cm^−1^, and 1231 cm^−1^, respectively, for each bacterium. The signal intensities increased linearly with the Log_10_ values of the concentrations (as shown in Figure 5B,D,F,H). The linear regression equations and the coefficients of determination (R^2^) are given in the captions of Figure 5B,D,F,H. The LODs for *S*. *aureus*, *S. typhimurium*, *E*. *coli* O157:H7, and *L*. *monocytogenes* were 5 CFU/mL, 4 CFU/mL, 23 CFU/mL, and 19 CFU/mL, respectively. The linear regression equations and the coefficients of determination (R^2^) for each bacterium are given with Figure 5B,D,F,H. Given the R^2^ values ranged from 0.956 to 0.998, there is a high correlation between the SERS signal intensity and the bacteria concentration. Consequently, it can be concluded this method achieved a high sensitivity detection of pathogenic bacteria in real beef samples.

Using AgNPs as the substrate, our technique can detect *E. coli* O157:H7 and *S. typhimurium* at lower concentrations than other SERS-based methods that used AuNPs-AgNPs conjugates [34]. Additionally, this technique has a lower LOD for *S. aureus* than other SERS-based methods that used AuNPs as the substrate [33], indicating that AgNPs are more effective than AuNPs for enhancing the Raman signals of *S. aureus*.

Alternatively, other SERS-based methods that used AuNPs as the substrate such as Zhang et al. [33] have lower LOD for *E. coli* O157:H7 in both pure culture and beef samples than the technique using AgNPs. This may be contributed to the higher Raman signal intensity of *E. coli* O157:H7 at 1320 cm^−1^ when AgNPs are used as the substrate. Similarly, other SERS-based methods that used AuNPs-AgNPs conjugates for example, Duan et al. [34] have lower LOD for *S. typhimurium* in both pure culture and beef samples than the current method. This may be attributed to the higher Raman signal intensity of *S. typhimurium* at 1337 cm^−1^ using AgNPs as the substrate. Yet a similar Raman signal intensity of *S. aureus* between 1300~1450 cm^−1^ has been noted when using AgNPs or Ag-Au as the substrate.

However, AgNPs technology cannot provide critical information such as bacterial activity and virulence, which affects the assessment of food microbial safety. Future studies need to optimize conditions, validate real samples, develop SERS portable systems, and evaluate bacterial characteristics in combination with other techniques.

### 3.4. LDA Results for the 4 Pathogenic Bacteria and Their Mixture in Beef Samples

As highlighted by Table 2, the model achieved high accuracy for all 4 individual pathogenic bacteria, with 100% correct rate for *E. coli* O157:H7, *S. aureus*, and *L. monocytogenes*, and 95% correct rate for *S. typhimurium*, which had only 5 spectra misassigned to *E. coli* O157:H7. LDA model can effectively discriminate between different pathogenic bacteria in beef samples based on their SERS spectra.

This has distinct advantages over other methods that used different techniques or algorithms for detecting or classifying bacteria in food samples. For example, Srividya et al. [35] used SERS combined with canonical discriminant analysis to classify *Mycobacterium tuberculosis* and *non-Mycobacterium tuberculosis*, and achieved 95% accuracy, which is lower than our average accuracy of 98.13%. This may be attributed to differences in the analysis as canonical discriminant analysis is less robust than LDA for handling high-dimensional data such as SERS spectra [36]. This study highlights LDA model can not only detect individual pathogenic bacteria, but also their mixture in beef samples, which is more challenging and realistic than detecting pure cultures or single species.

The current research limitation is that the LDA model only provides information on the presence of bacteria, and lacks data on quantity, growth rate and drug resistance, which are crucial for the assessment of food microbial safety. In addition, the study covered only four foodborne disease pathogens, and the complexity of the interaction between meat species and bacteria requires more research to address multiple meat/bacteria combinations.

### 3.5. The Practical Application Value and Potential Impact of the Research

This study utilized surface-enhanced Raman spectroscopy (SERS) technology to rapidly detect foodborne pathogenic bacteria in beef. It not only holds significant scientific importance but also has extensive potential value in practical applications.

#### 3.5.1. The Impact on Farmers

For farmers, rapid detection of pathogenic bacteria in meat can effectively reduce economic losses caused by pathogen contamination. By promptly detecting and controlling the spread of pathogens, farmers can better manage the health of their livestock, improve farming efficiency, and ensure the quality and safety of meat products. This not only helps increase farmers’ income but also enhances consumers’ trust in agricultural products [37].

#### 3.5.2. The Impact on Meat Processing Plants

Meat processing plants are subject to strict food safety standards and regulatory requirements during the production process. The rapid detection capabilities of SERS technology can significantly enhance production efficiency and reduce production delays caused by long testing times. Moreover, the high sensitivity and specificity of this technology can effectively detect low-concentration pathogenic bacteria, ensuring the safety of the products leaving the factory, reducing food safety risks, and thereby protecting the reputation and market competitiveness of the enterprise.

#### 3.5.3. The Impact on Beef Sellers and Sales Points

The beef sellers and sales points (such as supermarkets and butcher shops) need to ensure the safety of the sold meat during the sales process. The portability and rapid detection capability of the SERS technology make it an ideal on-site detection tool. By conducting rapid tests at the sales points, contaminated meat can be detected and dealt with promptly, preventing the sale of contaminated products to consumers, thereby reducing the occurrence of food safety incidents and protecting the health of consumers.

#### 3.5.4. The Impact on Family

Household consumers are the ultimate beneficiaries of food safety. Although households usually do not have professional testing equipment and techniques, the portability and ease of use of SERS technology make it possible to become a tool for household food safety testing. Through simple training, household consumers can use the portable Raman spectrometer to conduct rapid tests on meat before consumption, ensuring food safety and reducing health problems caused by consuming contaminated meat.

#### 3.5.5. The Impact on Catering Industry

The catering industry needs to strictly control food safety during the process of food processing and sales. The SERS technology can quickly detect pathogenic bacteria in meat, ensuring the safety of the ingredients. By conducting on-site tests in the kitchen, the catering industry can promptly identify and handle contaminated ingredients, preventing contaminated food from being provided to consumers, thereby protecting the health of consumers, reducing the occurrence of food safety incidents, and maintaining the reputation of the enterprise.

### 3.6. Future Directions

Based on the reliable methods and significant results of this study, surface-enhanced Raman spectroscopy (SERS) technology has demonstrated high sensitivity and accuracy in the rapid detection of foodborne pathogenic bacteria in beef, providing a new approach for food safety monitoring. However, this research also has some limitations. The SERS technology has high sensitivity and rapid detection advantages in detecting pathogens in food. However, in complex food matrices, such as beef, there may be some interfering factors. These interfering factors may include components like fat, protein, and pigment in the food, which may affect the SERS signal. Currently, the SERS technology is mainly based on the physical and chemical properties of bacteria for detection. Therefore, without additional biochemical methods for assistance, it is difficult to directly distinguish between live and dead bacteria. In future research, we can optimize the preparation method of the SERS substrate, select appropriate wavelengths for detection, and combine other technologies to improve the accuracy and reliability of the detection. Future research should focus on promoting the practical application and large-scale promotion of this technology, including developing portable, high-performance SERS detection devices, optimizing signal stability in complex food matrices, expanding to the detection and verification of more types of meat and pathogens, and integrating multiple technology fusion methods such as artificial intelligence to further improve detection efficiency and intelligence level, ultimately achieving the wide application and standardized deployment of this technology in the food industry.

## 4. Conclusions

Overall SERS was able to detect *E. coli* O157:H7, *S. typhimurium*, *S. aureus*, and *Listeria monocytogens* at different concentrations in both pure culture and beef samples. We established a prediction model for the 4 bacteria using LDA and calculated the recovery rate and the LOD of the SERS method. The average recovery rate was 99.47%, indicating that the SERS method is suitable for the detection of meat-borne pathogenic bacteria. For future work, we plan to optimize the SERS method for different types of meat samples and test its performance in real-world scenarios. However, it is important to acknowledge several limitations in the current study that need to be addressed for broader application. While beef is a significant component of meat consumption, the applicability of the SERS method to other types of meat, such as pork and poultry, remains untested. Future work should include testing the method on a wider range of meat types to validate its universal applicability.

## Figures and Tables

**Table 1:** Results of 4 pathogenic bacteria in beef samples by SERS detection method.

Bacteria	Concentration of Bacteria (lg(CFU/mL))	Detected Concentration of SERS (lg(CFU/mL))	Recovery Rate (%)	RSD (%)
*E. coli* O157:H7	1	0.91 ± 0.01	90.51	1.21
	2	2.21 ± 0.02	110.17	0.85
	3	3.03 ± 0.02	100.95	0.56
*S. typhimurium*	1	0.93 ± 0.02	92.98	2.37
	2	1.92 ± 0.02	96.00	1.24
	3	3.03 ± 0.03	100.97	1.00
*S. aureus*	1	0.91 ± 0.03	91.44	2.88
	2	2.18 ± 0.04	108.87	1.67
	3	3.04 ± 0.05	101.37	1.60
*L. monocytogenes*	2	2.15 ± 0.13	107.61	1.71
	3	2.90 ± 0.11	96.64	3.08
	4	3.84 ± 0.38	96.10	0.05

**Table 2:** LDA data of 4 pathogenic bacteria in beef samples.

Bacteria	Training Set (*n* = 240)						Test Set (*n* = 120)					
	*E*. *coli*	*STM*	*S*. *aur*	*LM*	Mixture of 4 Bacteria	Accuracy (%)	*E*. *coli*	*STM*	*S. aur*	*LM*	Mixture of 4 Bacteria	Accuracy (%)
*E. coli*	40	0	0	0	0	100	20	0	0	0	0	100
*STM*	4	36	0	0	0	90	1	19	0	0	0	95
*Saur*	0	0	40	0	0	100	0	0	20	0	0	100
*LM*	0	0	0	40	0	100	0	0	0	20	0	100
mixture of 4 bacteria	0	0	0	0	40	100	0	0	0	0	20	100
Overall accuracy						91.67						94.17
Average accuracy		92.92										

Note: *E. coli*: *E. coli* O157:H7; STM: *S. typhimurium*; *S. aur*: *S. aureus*; LM: L. monocytogenes.

## Data Availability

The original contributions presented in the study are included in the article, further inquiries can be directed to the corresponding author.
